# Pleuropulmonary blastoma in a 3-year-old child with persistent fever: a case report

**DOI:** 10.3389/fped.2025.1534277

**Published:** 2025-02-19

**Authors:** Ruiqi Yue, Guixia Ma, Li Mao, Qian Ni

**Affiliations:** ^1^ The Second Clinical Medical College of Lanzhou University, Lanzhou University, Lanzhou, China; ^2^ Pediatric Respiratory Medicine, Lanzhou University Second Hospital, Lanzhou, China

**Keywords:** pleuropulmonary blastoma, pediatric lung tumor, acute respiratory infections, misdiagnosis, DICER1 mutation

## Abstract

Pleuropulmonary blastoma (PPB) is a rare and highly aggressive malignant mesenchymal tumor, representing the most common malignant lung tumor in children. It is often misdiagnosed or underdiagnosed due to its clinical presentation, which mimics acute respiratory infections. Additionally, imaging manifestations are frequently indistinguishable from congenital respiratory malformations or severe lung infections. The tumor has high rates of recurrence and metastasis and has a poor prognosis. Currently, the internationally recommended treatment for PPB is a combination of surgery, chemotherapy, and radiotherapy. This article discusses the diagnosis and treatment of a 3-year-old girl with PPB combined with severe pneumonia.

## Highlights

• This study highlights the diagnosis and treatment of PPB in a 3-year-old girl initially misdiagnosed with bronchopneumonia.

• The methodology involved a comprehensive clinical evaluation, including chest imaging, laboratory tests, and multiple bronchoscopy procedures, followed by surgical exploration and genetic testing to confirm the diagnosis.

• Clinical practitioners ought to take into account the significance of differential diagnoses associated PPB in order to enhance the accuracy and timeliness of diagnosis and optimize the treatment strategy formulation for patients.

## 1 Introduction

Pleuropulmonary blastoma (PPB) is a highly aggressive primary malignant lung tumor. The diagnosis and treatment of the tumor require a multidisciplinary approach, involving specialists in imaging, pathology, thoracic surgery, and medical oncology who have experience in pediatric tumors. It is often associated with DICER1 gene mutations, and is the most common primary lung malignancy in children. It is prone to metastasize, predominantly to the brain parenchyma (1). PPB is extremely rare, with 25–50 cases reported annually in the USA (2). Its clinical manifestations, which may include fever, cough, sputum, and chest pain, often lead to misdiagnosis as respiratory infectious diseases like pneumonia or cystic adenoma of the lungs, or as congenital pulmonary airway malformations combined with respiratory infections. Pathologically, PPB is categorized as type I, II, or III. In type I PPB, adjuvant chemotherapy is usually not required (3). For type II and III PPB, systemic chemotherapy and surgical resection are critical. Early surgery can decrease child mortality, enhance prognosis, and extend survival; thus, early definitive diagnosis is crucial. This case highlights the difficulties and complexities in diagnosing lung tumors in children and provides insight into the clinical and diagnostic aspects of PPB, and provides a background on PPB.

## 2 Case report

**Figure 1:**
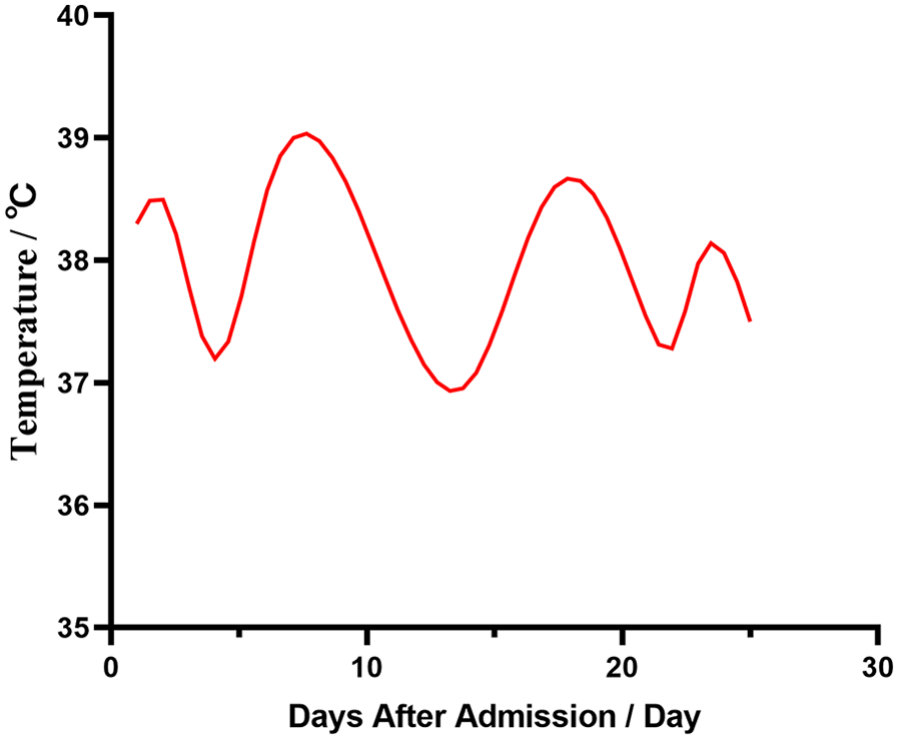
Changes in admission temperature.

A three-year-old female child was admitted to the hospital with bronchopneumonia at the outpatient clinic following a cough that worsened over four days, accompanied by a fever for three days. Before admission, she experienced coughing with yellow pus-like sputum and had fever peaks up to 39.3℃ occurring 2–3 times daily. She received “ceftazidime and peramivir” intravenously for two days; however, her fever and cough persisted, leading to her transfer to our hospital (Figure 1). Outpatient chest x-ray suggests pneumothorax.

**Figure 2:**
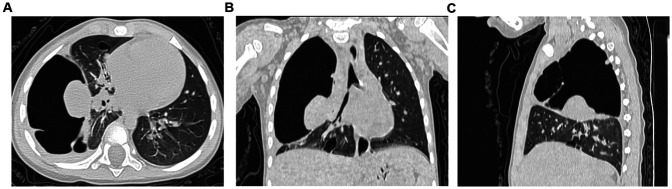
Chest CT plain scan performed on the day of admission. **(A)** Axial chest CT plain lung window; **(B)** Coronal chest CT plain lung window; **(C)** Sagittal chest CT plain lung window.

Upon admission, her temperature was 38.3 ℃, pulse 126/min, respiration rate 41/min, and blood pressure 83/51 mmHg. She appeared lethargic and weak. Auscultation revealed a few wet rales at the base of both lungs and diminished respiratory sounds in the upper right lung. Examinations of the heart and abdomen showed no abnormalities. No family history of malignant tumors or infectious diseases was reported. Antibody tests were positive for respiratory syncytial and parainfluenza viruses and weakly positive for Mycoplasma pneumoniae. Laboratory test results are presented in Table 1. Chest CT scans revealed uneven lung translucency and multiple cystic thin-walled translucent shadows in the right lung, the largest measuring approximately 52 × 76 × 89 mm. This scan also showed air-liquid levels with a small amount of pleural effusion on the right side (Figure 2). The initial diagnosis included bronchopneumonia, pneumothorax, pleural effusion, and congenital tracheal malformations. Initial treatment was given with piperacillin sodium tazobactam administered through the vein. The following day, thoracentesis was performed, draining 5–20 ml of hemorrhagic fluid daily via a closed chest drainage bottle.

**Table 1:** Comparison of infection indexes before and after 16 days of treatment in our children.

Admission time	The day of admission	Day 3 of admission	Day 16 of admission	Day 22 of admission	Normal reference values
WBC	14.2*10^9/L	13.1*10^9/L	7.2*10^9/L	7.2*10^9/L	4.40–11.90*10^9/L
NE%	0.57	0.55	0.43	0.8	0.22–0.65
LY%	0.30	0.32	0.45	0.6	0.23–0.69
MO%	0.13	0.10	0.10	0.03	0.02–0.11
RBC	3.80*10^12/L	4.05*10^12/L	4.24*10^12/L	2.61*10^12/L	4.00–5.00*10^12/L
HGB	103 g/L	108 g/L	110 g/L	72 g/L	112–149 g/L
PLT	316*10^9/L	662*10^9/L	158*10^9/L	388*10^9/L	188–472*10^9/L
CRP	145.50 mg/L	17.68 mg/L	<9.99 mg/L	<9.99 mg/L	<10.00 mg/L
hs-CRP	>10.00 mg/L	>10.00 mg/L	<0.80 mg/L	<3.00 mg/L	<3.00 mg/L
SAA	252.13 mg/L	17.33 mg/L	<4.80 mg/L	11.20 mg/L	<10.00 mg/L
FIB	4.32 g/L	3.66 g/L	1.48 g/L	1.48 g/L	2.00–5.00 g/L
Dimer	3.70 ug/ml	2.32 ug/ml	1.76 ug/ml	1.39 ug/ml	<0.5 ug/ml
FDP	6.49 ug/ml	4.33 ug/ml	3.01 ug/ml	2.54 ug/ml	<5.00 ug/ml
PCT	6.500 ng/ml	0.111 ug/ml	0.059 ng/ml	1.010 ng/ml	0–0.046 ng/ml
ALB	32.2 g/L	/	/	31 g/L	39.0–54.0 g/L
CK-MB	21 U/L	/	/	52 U/L	<24 U/L
LDH	303 U/L	/	/	392 U/L	120–250 U/L

WBC, white blood cell count; NE%, neutrophil percentage; LY%, lymphocyte percentage; MO%, monocyte percentage; RBC, red blood cell count; HGB, hemoglobin concentration; PLT, platelet count; CRP, C-reactive protein; hs-CRP, high sensitivity C-reactive protein; SAA, serumamyloid A; FIB, fibrinogen; Dimer, D-Dimer; FDP, fibrin degradation Product; PCT, calcitoninogen; ALB, albumin; CK-MB, serum creatine kinase isozyme; LDH, lactate dehydrogenase.

**Figure 3:**
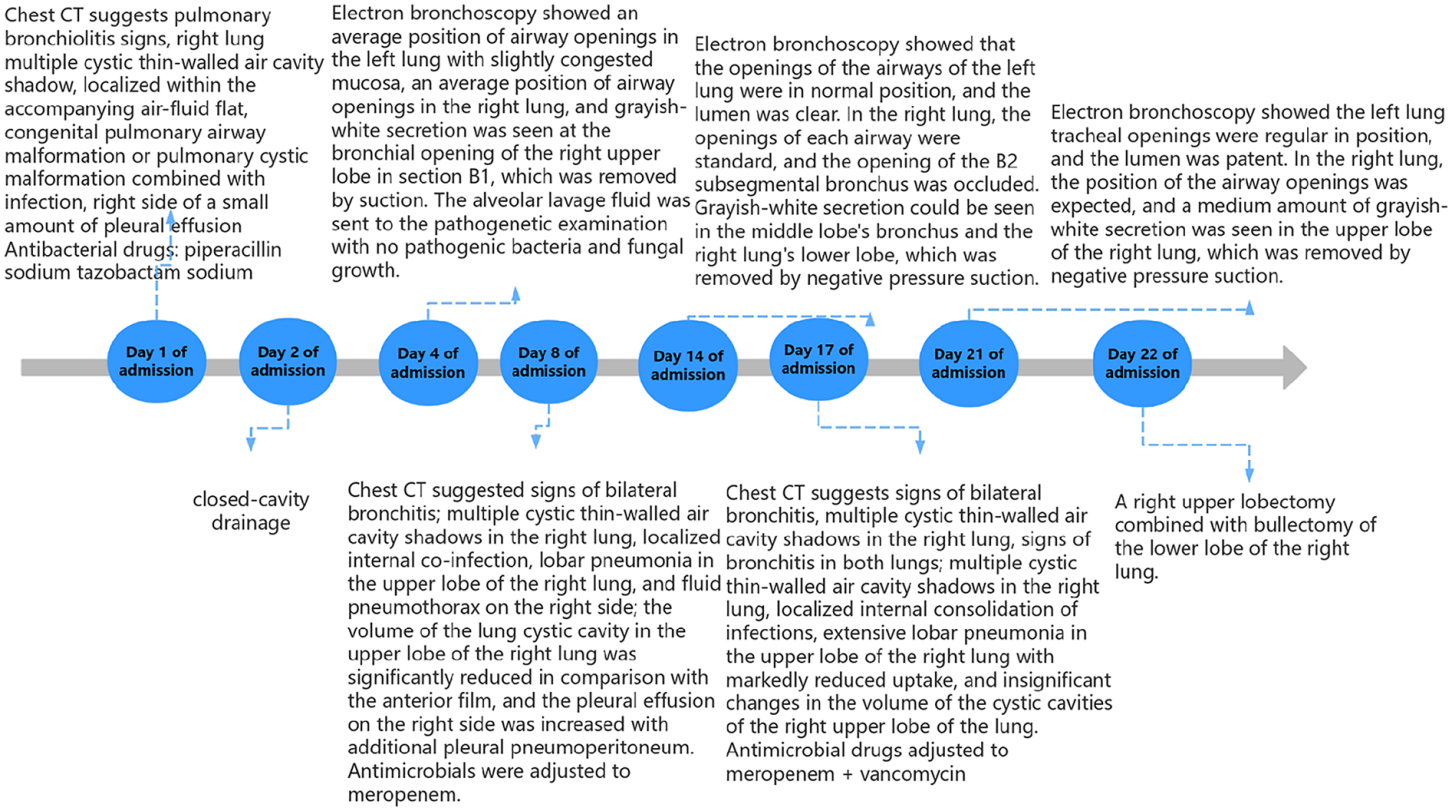
Flowchart of the hospitalization process.

**Figure 4:**
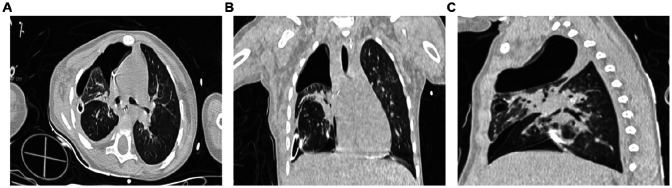
Chest CT scan on the first postoperative day. **(A)** Axial chest CT plain lung window; **(B)** Coronal chest CT plain lung window; **(C)** Sagittal chest CT plain lung window.

An electronic bronchoscopy was conducted on day four of hospitalization, as shown in Figure 3. Anti-infective treatments don't seem to have much effect. laboratory examination in Table 1. Chest CT revealed multiple cystic, thin-walled, translucent shadows in the right lung and flaky, high-density shadows in the upper lobe; fluid density and gas shadows were observed in the right thoracic cavity (Figure 3). The treatment regimen was adjusted to meropenem. Subsequently, although the patient's body temperature exhibited a decline compared to previous levels, it still failed to return to normal ranges. To alleviate the symptoms, on admission day 14, a second electronic bronchoscopy was performed (results shown in Figure 3). Infection indicators are listed in Table 1. Chest CT indicated signs of a significant decrease in the absorption of lobular pneumonitis in the right upper lobe and no significant change in the volume of the right lung cystic cavity. Reductions in drainage and pleural effusion were observed compared to the previous scans, although the size of the lesion remained unchanged. For further temperature control, vancomycin was added to the treatment regimen without effective temperature control on day 16. A third electronic bronchoscopy was performed on day 21, with results in Figure 3. After consulting with the thoracic surgery department and analyzing treatment outcomes, a decrease in infectious indices was noted, but persistent fever remained, potentially linked to a developmental congenital pulmonary cyst. The team decided on surgical exploration, which included a right upper lobectomy combined with bullectomy of the lower lobe of the right lung. The postoperative chest CT results are shown in Figure 4. Pathological diagnosis confirmed a right upper lobe PPB (type II) and a right lower lobe congenital pulmonary airway malformation (type IV). Genetic testing identified a DICER1 gene mutation. The parents then transferred the child to an out-of-town hospital for chemotherapy.

## 3 Discussion

PPB is a clinically rare pediatric thoracic malignant tumor, composed of malignant stromal cells and primitive blastoid cells (2). PPB predominantly affects children, often presenting nonspecific symptoms such as fever, cough, sputum, chest pain, or bronchopneumonia accompanied by pneumothorax and pleural effusion (4, 5). The disease is frequently misdiagnosed as pneumonia or congenital cystic adenomatoid malformation of the lung. The exact pathogenesis remains unclear, but a genetic basis has been identified in about 40% of cases. Patients with PPB often have mutations in the DICER1 gene (6, 7).Early testing for this gene can assist in clarifying the diagnosis (8).In approximately 10% of cases, PPB may coexist with multilocular cystic nephroma and occasionally with nephroblastoma. It has been reported that PPB may develop from congenital lung cysts, with the rate of these cysts developing into malignant tumors at 8.6% (9).Furthermore, a study by Vargas et al. noted chromosome 8 polymorphism as a persistent genetic aberration in PPB (10, 11). PPB is prone to metastasis, with the central nervous system as the most frequent site for extrathoracic metastasis. The tumor can also spread to other organs, including bones, liver, pancreas, kidneys, and adrenal glands (12–14).

PPB is pathologically categorized into type I, type II, and type III. Type I is cystic; type II is cystic-solid, often with liquefaction, necrosis, and hemorrhage; and type III is solid (15, 16).As malignancy increases, the solid component of the tumor also increases. Type II and III lesions frequently involve the pleura extensively and causing pleural effusion (11). Imaging of PPB typically shows non-specific features. It usually occurs unilaterally, predominantly in the upper lobe of the right lung (17), often visible on chest radiographs. CT scans often reveal a large, isolated, solid mass, sometimes with cystic degeneration or necrosis. PPB rarely connects with the trachea and bronchi, making it difficult to detect via e-bronchoscopy.

The non-specific clinical and imaging presentations of PPB make it easy to overlook or misdiagnose. Li Xiaobing et al. (18) noted that 5 out of 6 patients were initially misdiagnosed: three with pneumonia, one with pleural effusion, and one with congenital cystic adenomatoid malformation (CCAM); Nars et al. (19) identified 2.3% of 129 CCAM cases as type I PPB post-surgery. This report focuses on a child initially diagnosed with severe pneumonia and tracheal malformation. The child's minor hemorrhagic pleural fluid, which was initially thought to result from puncture injuries, was overlooked as a potential sign of carcinomatous pleural fluid. The diagnosis was confirmed through surgical exploration and histopathology.

Diagnosis and treatment of PPB currently face significant challenges. Early improvements in diagnostic techniques are crucial for reducing the rates of missed and misdiagnoses and may provide patients with an opportunity for long-term survival. Determining appropriate surgical plans and the correct use of radiotherapy and chemotherapy are vital. Surgery is the primary treatment, with enlarged resection and lymph node dissection as the common approach. Adjuvant chemotherapy or radiotherapy usually follows. Platinum-based chemotherapy suits some cases but varies individually. Currently, no optimal chemotherapy regimen exists.

This case report has several limitations. First, there is a lack of generalizability due to differences in case volumes and mixes. The child was transferred to out-of-hospital chemotherapy, it was not possible to clarify a causal relationship between the chemotherapy regimen in question and the condition and prognosis (20).

During the diagnosis and treatment of this case, we've gained the following insights: For children with unexplained recurrent fever and cough, whose infection index stays high without symptom relief even after improved anti-infective therapy, and when CCAM and multiple cystic lung lesions are detected by chest CT, we should be alert to the possibility of PPB. Moreover, continuous monitoring and follow-up are essential in such situations.

## 4 Conclusion

PPB is a rare lung malignancy seen in children. It has high rates of recurrence and metastasis and is associated with poor prognosis. While the preferred treatment is surgery, there is no unified treatment plan. Due to the lack of specificity of its clinical manifestations and atypical imaging features, clinical diagnosis of PPB is particularly difficult. This report discusses the issues involved in clinical diagnosis and provides ideas and strategies for the treatment of PPB.

## Data Availability

The original contributions presented in the study are included in the article/Supplementary Material, further inquiries can be directed to the corresponding author.
